# Efficient Bioremediation of Petroleum-Contaminated Soil by Immobilized Bacterial Agent of *Gordonia alkanivorans* W33

**DOI:** 10.3390/bioengineering10050561

**Published:** 2023-05-08

**Authors:** Yong Yang, Wanze Zhang, Zhanwei Zhang, Ting Yang, Zhuo Xu, Chuanbo Zhang, Bing Guo, Wenyu Lu

**Affiliations:** 1 School of Chemical Engineering and Technology, Tianjin University, Tianjin 300072, China; yangyong@cnooc.com.cn (Y.Y.); zhangwanz@126.com (W.Z.); zwzspace@tju.edu.cn (Z.Z.); luxidia@163.com (Z.X.); zcbtju@tju.edu.cn (C.Z.); 2 CNOOC EnerTech-Safety & Environmental Protection Co., Tianjin 300457, China; 3 China Offshore Environmental Service Ltd., Tianjin 300457, China; yangting11@cnooc.com.cn (T.Y.); guobing5@cnooc.com.cn (B.G.)

**Keywords:** bioremediation, microbial immobilization, *Gordonia alkanivorans* W33, petroleum-contaminated soil

## Abstract

In this article, we report a method for preparing an immobilized bacterial agent of petroleum-degrading bacteria *Gordonia alkanivorans* W33 by combining high-density fermentation and bacterial immobilization technology and testing its bioremediation effect on petroleum-contaminated soil. After determining the optimal combination of MgCl_2_, CaCl_2_ concentration, and culture time in the fermentation conditions by conducting a response surface analysis, the cell concentration reached 7.48 × 10^9^ CFU/mL by 5 L fed-batch fermentation. The W33-vermiculite-powder-immobilized bacterial agent mixed with sophorolipids and rhamnolipids in a weight ratio of 9:10 was used for the bioremediation of petroleum-contaminated soil. After 45 days of microbial degradation, 56.3% of the petroleum in the soil with 20,000 mg/kg petroleum content was degraded, and the average degradation rate reached 250.2 mg/kg/d.

## 1. Introduction

During the use, transportation, loading, and unloading of petroleum, the environment will be polluted because of improper operation [1,2]. Due to frequent accidents and human activities, soil pollution caused by petroleum and its chemical products has become an environmental problem worldwide that cannot be ignored [3,4,5]. The concentration of total petroleum hydrocarbons (TPH) in contaminated soil varies from trace levels to 236,500 mg/kg according to the degree of pollution [6,7,8]. An economical, efficient, and environmentally friendly disposal method is required to reduce the harm caused by petroleum-contaminated soil [9,10]. Compared with physical and chemical methods, the bioremediation method is not only cheap to apply, but also completely repaired, without secondary pollution to the environment, and has a very good prospect for promotion [11,12,13].

At present, a large amount of the literature has reported the application of biological methods to repair petroleum-contaminated soil [14,15,16], and a variety of biological treatment methods have been analyzed, including biological stimulation (by adding nutrients to stimulate the degradation of indigenous petroleum-degrading microorganisms) and biological enhancement (by adding exogenous petroleum to degrade microorganisms to enhance the degradation of petroleum) [17,18,19,20]. Hafida et al. reported that the maximum TPH removal reached 55% in non-sterilized soil at an initial concentration of 2% after 28 days of *Streptomyces* sp. Hlh1 incubation [3]. Bidja Abena et al. found the addition of exogenous bacteria increased the removal of TPH in a highly contaminated soil sample. The concentration of TPH in that soil was reduced from 236,500 mg kg^−1^ of dry soil to 176,566 mg kg^−1^ of dry soil in 40 days, which was equivalent to 25.4% degradation of TPH [6]. Ramadass et al. added inorganic N, P, and K components to petroleum hydrocarbon-contaminated soil to achieve the purpose of biological stimulation. After 210 days of biodegradation, 74% of the engine oil was removed [21]. For 600 m^3^ of petroleum-contaminated soil, Beškoski VP et al. carried out a 150-day ectopic bioremediation test and found that the degradation rate of alkanes was the fastest, which was 23.7 mg/kg/d; the aromatic hydrocarbon was the second, which reached 5.7 mg/kg/d; the asphalt components had the slowest biodegradation rate, which was 3.3 mg/kg/d [22]. Although some progress has been made in the study of microbial repair, biological repair still has the disadvantages of a long processing time and low oil bioavailability. Increasing the concentration of petroleum-hydrocarbon-degrading bacteria in the environment and enhancing the environmental adaptability of microorganisms have become the key to the bioremediation of petroleum-contaminated soil.

During the bioremediation process, the density of petroleum hydrocarbon-degrading bacteria has an important effect on pollution remediation. A higher concentration of bacteria can increase the catalytic activity of related enzymes and speed up the process. In addition, the immobilization technology of microorganisms can improve the removal rate of TPH by maintaining cell viability [11] or improving mass transfer [23]. Kureel et al. used polyurethane foam as a carrier to immobilize a *Bacillus* strain and subsequently treated benzene-contaminated soil in a packed bed reactor. The results indicated that the benzene-removal rate of immobilized microorganisms reached 90% in 9 days, which was 20% higher than that of free microorganisms [24]. Xiong et al. used biochar as a carrier to immobilize a mycobacterium that can degrade polycyclic aromatic hydrocarbons. The results showed that after 18 days of degradation, the free bacteria could hardly degrade fluoranthene and benzopyrene in the soil, while the removal rates of both fluoranthene and benzopyrene were over 50% after immobilization [25]. Hale et al. inoculated immobilized microorganisms and free microorganisms with biochar as carriers into soil. By measuring the number of microorganisms in the soil, they found that compared with the inoculant-free microorganisms, the survival rate of the immobilized bacteria was significantly improved [26].

**Figure 1:**
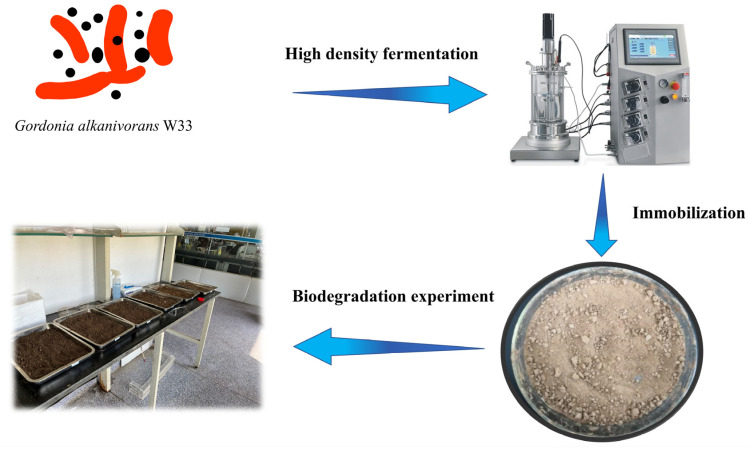
Scheme of this study.

In this study, we combined high-density fermentation with microbial immobilization to improve the density of petroleum-hydrocarbon-degrading bacteria (Figure 1). During high-density fermentation, some feed solution was added to the medium to provide a more excellent growth environment for the microorganisms, often including inorganic salts and vitamins [27,28,29]. Here, we selected some inorganic salts, MgCl_2_, MnCl_2_, CaCl_2_, FeSO_4_ and (NH_4_)_2_SO_4_, as stimuli and added them separately to the culture medium. The results showed that adding MgCl_2_ or CaCl_2_ alone was effective (Figure S1a). Therefore, the concentrations of MgCl_2_ and CaCl_2_ were selected as key factors affecting fermentation. We used the response surface method to optimize the fermentation conditions of *Gordonia alkanivorans* W33 (W33) to obtain a higher bacteria concentration, and the results showed that the bacteria concentration increased by 6.62 folds. The mix of vermiculite powder with sophorolipids and rhamnolipids to immobilize W33 not only improved the survival rate of the cells inoculated in the soil but also accelerated the degradation of petroleum. Approximately 56.3% of the petroleum in the soil was removed in only 45 days. In addition, this bioremediation test proved that W33 has good prospects for large-scale application.

## 2. Materials and Methods

### 2.1. Chemicals, Soil, Biocarrier, and Biosurfactant

All of the solvents used were analytical reagents (purity 99.7%), obtained from Tianjin Guangfu Fine Chemical Research institute. The vermiculite powder was purchased from Guangning Mineral Processing Factory, Lingshou, Hebei, China. The rice husk was purchased from Songzhuo Agricultural Byproduct Sales Co., Ltd., Shijiazhuang, Hebei, China. The sawdust was purchased from Jixi Mining Products Co., Ltd., Lingshou, Hebei, China. The diatomaceous earth was purchased from Jushi Mineral Product Processing Factory, Lingshou, Hebei, China. The activated carbon was purchased from Kaibiyuan Trading Co., Ltd., Beijing, China. The straw was purchased from Mufeng Planting Farmers’ Professional Cooperative, Tangshan, Hebei, China. The petroleum was supplied by CNOOC EnerTech-Safety & Environmental Protection Co. The strain used in this study is *G. alkanivorans* W33 petroleum-hydrocarbon-degrading bacteria preserved in the laboratory. The isolation, screening, and identification of *G. alkanivorans* W33 were completed in the previous work of Chen Yu [30]. In Chen Yu’s previous work, the degradation rate of W33 on 1% petroleum reached 91.73% after 9 days of degradation.

The unpolluted soil (pH = 8.2 and water content = 0.3183 g/g) was collected from the coastline in Tian Jin Binhai New Area (117.8°/E, 39.0°/N), which belonged to Solonchacks. When collecting the soil, the topsoil was removed and 10–20 cm of the soil was collected below the surface. The soil was homogenized and passed through a 2 mm sieve. The soil was naturally air-dried and then mixed with petroleum on the premise that the petroleum concentration was 20,000 mg/kg soil and stirred well [6,21].

Sawdust (pH = 7.0), rice husk (pH = 6.9), diatomaceous earth (pH = 7.8), straw (pH = 7.3), activated carbon (pH = 8.5), and vermiculite powder (pH = 7.6) were selected as six immobilized carriers [31,32,33,34,35]. The carrier materials were filtered through a 200-mesh sieve (aperture 74 μm and 200 meshes per square inch), dried in an oven at 55 °C for 3 h, then laid flat and irradiated with ultraviolet light on a clean bench for 8 h before being used.

Sophorolipids and rhamnolipids were used as biosurfactants on the surface of the immobilized W33 bacterial agent, in order to reduce the surface tension of petroleum and improve the mass transfer between petroleum and bacteria. Lu et al. found that the best weight ratio of petroleum to sophorolipids + rhamnolipids is 1:0.25, and the weight ratio of 9:10 is the best composition of rhamnolipids and sophorolipids to minimize the surface tension of petroleum. Therefore, in this study, we used a mixture of rhamnolipids and sophorolipids with a total weight of 5 g and a weight ratio of 9:10 (2.4 g of rhamnolipids and 2.6 g of sophorolipids) as one of the experimental groups [36].

### 2.2. Cultivation of Bacteria

*Gordonia alkanivorans* W33 was cultivated in the following mediums. Inclined medium (1 L): tryptone 17.0 g, soy peptone 3.0 g, NaCl 5.0 g, K_2_HPO_4_ 2.5 g, glucose 5 g, and agar powder 20 g (TSB medium). The seed medium and the initial fermentation medium are the above formulations of removing the agar component. Feed medium (1 L): tryptone 60 g, soy peptone 15 g, K_2_HPO_4_ 10 g, glucose 50 g, MgCl_2_ 0.067 g, and CaCl_2_ 0.114 g and the balance was water. All of the mediums above were sterilized at 115 °C for 25 min.

### 2.3. Optimization of the Fermentation Conditions of G. alkanivorans W33

#### 2.3.1. Single Factor Test for the Medium

A single clone was picked from the TSB plate and inoculated into a 250 mL shake flask containing 50 mL of culture medium and cultured as a seed solution for 24 h. The seed solution was then diluted into a new 250 mL shake flask containing different concentrations of MgCl_2_ (0, 0.03, 0.06, 0.09, and 0.12 mM) and different concentrations of CaCl_2_ (0, 0.03, 0.06, 0.09, and 0.12 mM) in TSB medium, at an initial OD_600_ of 0.5, and then cultured at 200 rpm at 35 °C. The biomass in the fermentation broth was then measured at a wavelength of 600 nm using an ultraviolet-visible spectrophotometer.

#### 2.3.2. Box–Behnken Center Combination Design Experiment

According to the results of the single-factor optimization experiment, the final biomass (Y) of the bacteria W33 was used as the response value, and the effects of fermentation time (A), MgCl_2_ concentration (B), and CaCl_2_ concentration (C) on the final biomass (Y) of the fermentation were investigated. A three-factor, three-level Box–Behnken test was designed using Design Expert 10.0.4. The response surface model factors and level design are shown in Table S1.

### 2.4. 5 L Fermenter Fermentation

Batch fermentation: The cultured seed solution was inoculated at a ratio of 10% in a 5 L fermenter containing 3 L medium, with a controlled speed of 220 r/min and aeration of 3 L/min. The dissolved oxygen was maintained above 40%. Then, in the case where the initial pH of the fermentation broth was 7.2, fermentation was carried out for 87 h.

Fed-batch fermentation: The cultured seed solution was inoculated at a ratio of 10% in a 5 L fermenter containing 3 L medium, with a controlled speed of 220 r/min. The dissolved oxygen was maintained above 40%, and aeration of 3 L/min. The initial pH was about 7.2; at the 30th hour of fermentation, the feed medium was added at a rate of 1 mL/min to control the sugar concentration to less than 2 g/L, and a total of 1.5 L was fed.

### 2.5. Immobilization of the Bacteria and Detection of the Immobilized Consequent

After the culture was completed, the fermentation broth was centrifuged and washed three times with a phosphate buffer (pH 7.0). The bacteria were resuspended in a volume of Bushnell–Haas (BH) medium containing 2% glucose equal to the fermentation broth [37], and then they were mixed with the biocarrier in a ratio of 1:1 (mL:g). The carrier culture was incubated at 35 °C for 3 days and then placed in a sterile hood for use. To the experimental groups of the immobilized bacterial agent with the biosurfactant 5 g of lactone-type sophorolipids or a 5 g mixture of rhamnolipids and lactone-type sophorolipids was added after completing the above steps and then stirred well.

Approximately 1 g of the immobilized bacterial agent was taken to be resuspended in 100 mL of 0.9% NaCl solution, and the mixture was stirred in a shaker for 10 min to suspend the adhered cells. The resulting supernatant was serially diluted and spread on TSB agar plates. The colonies were counted after 3 days of incubation in a 35 °C incubator.

### 2.6. Biodegradation of the Petroleum-Contaminated Soil

Approximately 1 kg simulated contaminated soil was spread in a steel tray with a size of 40 cm × 30 cm × 4.8 cm. Approximately 20% (kg/kg) of the immobilized bacterial agent or the W33 bacterial solution containing the same number of cells was added to the simulated contaminated soil. In this study, five biodegradation groups were set, including the simulated contaminated soil with the W33 bacteria solution added, the simulated contaminated soil with the W33-immobilized bacterial agent added, the simulated contaminated soil with the W33-immobilized bacterial agent and sophorolipids added, the simulated contaminated soil with the W33-immobilized bacterial agent and glycolipid mixture added, and the simulated contaminated soil as the control group. The simulation devices were placed in an indoor ventilated place with all parameters set. A small amount of water was added with a watering can to each group every morning to maintain the normal growth and metabolism of the microorganisms.

### 2.7. Determination of Petroleum Content in the Contaminated Soil

A series of petroleum standard solutions were prepared. Petroleum can tightly bind with soil colloids and some organic compounds to form complex complexes, and it has to be extracted using organic solvents with strong polarity [38]. Approximately 1000.0 mg of petroleum was accurately weighed in a 100 mL beaker and 50 mL of CH_2_Cl_2_ was added to dissolve it; then, the petroleum-CH_2_Cl_2_ solution in the beaker was transferred to a 1000 mL volumetric flask, and CH_2_Cl_2_ was added to the 1000 mL mark.

A series of petroleum-CH_2_Cl_2_ solutions were prepared as required. Approximately 1 mL, 2 mL, 4 mL, 6 mL, and 8 mL of the 1000 mg/L petroleum-CH_2_Cl_2_ standard solution were added to a 100 mL volumetric flask, and CH_2_Cl_2_ was added to the 100 mL mark. Approximately 3 mL quartz cuvettes were used to hold a series of petroleum-CH_2_Cl_2_ standard solutions. Both oils and oil products do have certain characteristic absorbances in the UV range [39,40], and there have been many reports using ultraviolet spectrophotometry to determine the content of petroleum [16,40,41,42]. The UV-visible spectrophotometer (MAPADA UV-1200 spectrophotometer, Shanghai Mapada Instruments Co. Ltd., Shanghai, China) was used to detect the absorbance of each quartz cuvette with the petroleum-CH_2_Cl_2_ standard solution. A full scan of the petroleum-CH_2_Cl_2_ standard solution was carried out to obtain the appropriate wavelengths, and the concentrations of petroleum were measured by a UV-visible spectrophotometer at the maximum wavelength of 254 nm. The standard curve was drawm, with the absorbance as the ordinate and the concentration of the standard solution as the abscissa.

For all five test groups, the petroleum content was determined by the following method. Approximately 5 g of the dried soil sample was weighed accurately, and then it was loaded into a 50 mL centrifuge tube. Then, 40 mL of CH_2_Cl_2_ was added to each centrifuge tube for the extraction. The centrifuge tube was shaken for 1 min; then, the centrifuge tube was placed in an ultrasonic scrubber for extraction for 15 min; centrifugation took place at low speed (4000 r/min) for 8 min; the extract was filtered from the centrifuge tube into a 100 mL volumetric flask with filter paper. The above operation was repeated again; the extract was collected into the same volumetric flask and CH_2_Cl_2_ was added to the 100 mL mark. Approximately 5 mL of the above solution was taken to a 25 mL volumetric flask with a pipette and diluted to the 25 mL mark with CH_2_Cl_2_; then, 3 mL of it was taken to determine the absorbance in the quartz cuvettes. The petroleum content in the 5 g sample was calculated from the standard curve. The total petroleum content in the steel tray was obtained by:

Petroleum content of the 5 g sample (mg) × (Weight (g) of petroleum-contaminated soil + Weight (g) of immobilized bacterial agent)/5 g

### 2.8. Observation of the Contaminated Soil by an Electron Microscope

Instrument model: Czech TESCAN MIRA LMS. Gold sputtering target: Pt. Electron gun: Schottky Field Emission Electron Gun. Resolution: 0.9 nm @ 15 Kv (Secondary electron image); 2.0 nm @ 30 Kv (backscattered electron image). Electron optical path: the electron beam in the lens barrel has no cross path. Acceleration voltage: 200 v–30 kv. Probe beam current: 1 pA–100 nA, with a stability better than 0.2%/h. Magnification: 8×–100,000×. Objective lens: electromagnetic/electrostatic compound lens. Detector: in-beam SE and SE secondary electron detector, backscatter detector, and EDS spectrometer. Spectrum model: Xplore 30. Energy spectrum analysis working distance: 15 mm. Sample Stage Stroke: X = 125 mm; Y = 125 mm; Z = 50 mm; T = −60° to 60°; and R = 360° (continuously adjustable). Image capture: up to 16 k × 16.

Take a small amount of the sample/block/film and stick it directly to the conductive adhesive and use the Oxford Quorum SC7620 sputtering coater to spray gold for 45 s (the specific gold spraying time is determined according to the sample/test requirements), and the gold spraying is 10 mA. Then use TESCAN MIRA LMS. The sample morphology and energy spectrum mapping were tested by a scanning electron microscope. The accelerating voltage was 3 kV when the morphology was photographed, and the accelerating voltage was 15 kV when energy spectrum mapping was taken. The detector was an SE2 secondary electron detector.

The soil samples were taken from each group of steel trays at 45 days, with a mass of 10 g.

## 3. Results

### 3.1. Shake Flask Fermentation for the Single-Factor Experiment

Optimization of the fermentation conditions, such as the type and content of the medium carbon source and the nitrogen source and optimization of the initial inoculum volume, pH, culture temperature, and stirring speed were reported in Chen Yu’s work [30]. They concluded that the TSB medium with 17 g/L peptone, 2.5 g/L glucose, 5 g/L NaCl, and 2.5 g/L K_2_HPO_4_ was the optimal medium formula; a culture temperature of 35 °C, a culture time of 78 h, an initial inoculum volume of 10%, and a shaker rotation speed of 180 r/min are the best shake flask fermentation conditions [30]. Here, we mainly explore whether there are other factors that can increase the bacterial concentration to achieve a higher W33 fermentation density on this basis. The key factors were screened by a single-factor design experiment.

There are more and more studies on the mechanism of action of Ca^2+^ and Mg^2+^ in prokaryotic cells [43,44,45,46,47]. In this study, the change in the biomass of W33 with the concentration of Ca^2+^ and Mg^2+^ and time is shown in Figure S1b,c. It can be clearly observed that in the course of the change in the concentration of the two ions from 0 to 0.15 mM, the ultimate biomass increased first and then decreased, reaching a maximum at 0.06 mM Ca^2+^ and 0.09 mM Mg^2+^, respectively. With the change in culture time, it conforms to the “S” curve and reaches the stable phase at 80 h. From the growth curve in the TSB shake flask culture shown in Figure S1d, it can be seen that W33 begins to enter the logarithmic growth phase at around 20 h and reaches its maximum biomass at 80 h. The optimal culture conditions for W33 were determined as 0.09 mM MgCl_2_, 0.06 mM CaCl_2_, and culturing for 80 h by the single-factor optimization experiments. Next, the response surface test was used to examine the interaction between the three.

### 3.2. Box–Behnke Optimization

The response surface test plan was designed, and the results are shown in Table S2.

Using Design Expert 10.0.4 statistical analysis software, the regression equation of the biomass (Y) obtained by fitting the three obtained factors is:

Y = 45.96 + 2.31 A + 3.93 B + 2.41 C + 0.35 AB − 1.68 AC − 0.25 BC − 5.79 A^2^ − 1.87 B^2^ − 1.44 C^2^

Regression analysis was used to analyze the variance between each factor of the equation and the equation. The results are shown in Table S3, which shows that the model is extremely significant (*p* < 0.01). The model’s decision coefficient R^2^ = 0.9700, and its adjustment determination coefficient R^2^_Adj_ = 0.9314, indicating that the equation fits well to the test and this model can be used to predict and analyze response values. The order of influence of each factor on the final biomass is B > C > A, which means MgCl_2_ concentration > CaCl_2_ concentration > fermentation time. The concentration of Mg^2+^ and Ca^2+^ and the fermentation time had a significant effect on the final biomass (*p* < 0.01 for each quadratic term). The interaction between the fermentation time and the calcium chloride concentration was significant (0.01 < *p* < 0.05). On the contrary, the interaction between the fermentation time and the MgCl_2_ concentration and the interaction between the MgCl_2_ concentration and the CaCl_2_ concentration was not significant (0.05 < *p*).

**Figure 2:**
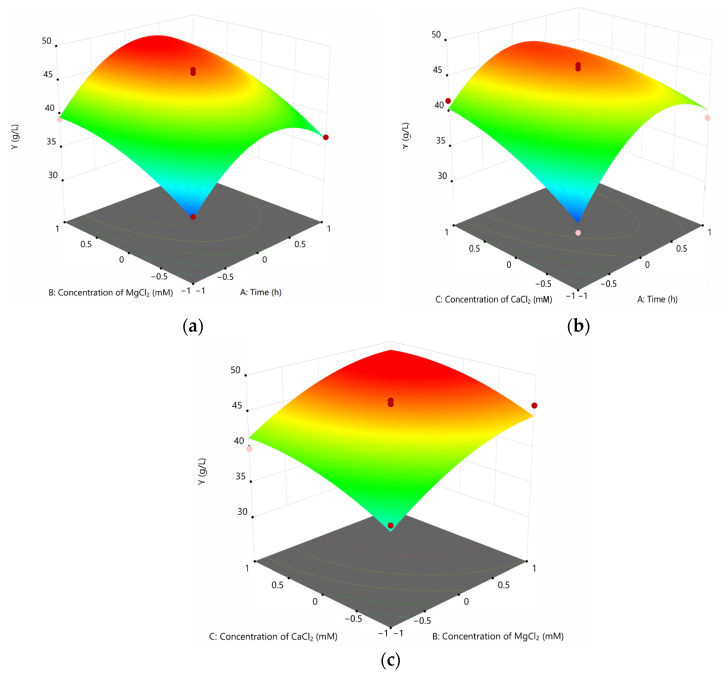
Response surface diagram of the interaction of various factors with the W33 biomass: (**a**) effect of the interaction between the concentration of MgCl_2_ and culture time on the biomass of W33; (**b**) effect of the interaction between the concentration of CaCl_2_ and culture time on the biomass of W33; and (**c**) effect of the interaction between the concentrations of CaCl_2_ and MgCl_2_ on the biomass of W33.

Considering the influence of the interaction of the other two factors on the final biomass under the condition that two factors are fixed at the same central value, a three-dimensional response surface was drawn. The result is shown in Figure 2.

The best set of process parameters predicted by the software were: 80 h fermentation time, 0.012 mM MgCl_2_, and 0.068 mM CaCl_2_, with a predicted maximum biomass of 46.024 mg/mL. After the verification test, the average biomass was 46.1 mg at the end of fermentation (Figure S2), indicating that the model predicted the actual fermentation well. The optimized fermentation broth was subjected to measurement of the fermentation unit by the plate colony counting method. The number of viable cells was 3.2 × 10^9^ CFU, which was 6.62-fold higher than that of 4.2 × 10^8^ CFU before optimization.

### 3.3. 5 L Fermenter Fermentation

**Figure 3:**
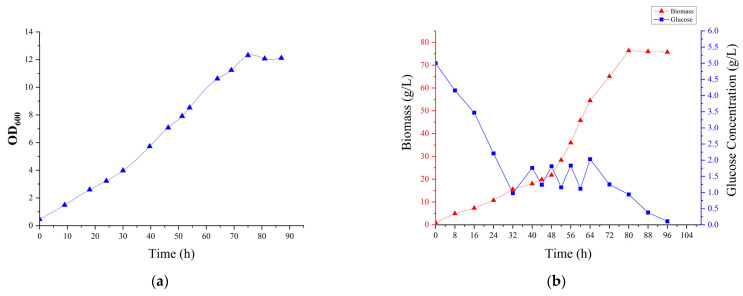
Fermentation curve of bacteria W33. (**a**) OD_600_ growth curve of bacteria W33 in batch fermentation and (**b**) fed-batch fermentation growth curve of bacteria W33. The data were the average of three measurements.

We first performed 5 L batch fermentation. MgCl_2_ and CaCl_2_ were added to the medium to the final concentration of 0.12 mM and 0.068 mM, respectively. The growth curve of W33 on the 5 L tank was obtained by sampling the fermentation from the 5 L tank. As shown in Figure 3a, the highest biomass was obtained at 75 h with the OD_600_ = 12.307, and the colony number reached 2.72 × 10^9^ CFU.

We then investigated the growth of W33 in 5 L fed-batch fermentation. MgCl_2_ and CaCl_2_ were added to the medium to the final concentrations of 0.12 mM and 0.068 mM, respectively. The biomass of the fermentation broth was measured every 8 h before feeding. During feeding, the biomass of the fermentation broth was measured every 4 h. After feeding was completed, the biomass of the cells was measured every 8 h until 96 h. The growth curve is shown in Figure 3b. At the end of fermentation, 1 mL of the fermentation broth was taken to determine the biomass. The weight reached 76.35 mg and the number of viable cells obtained by plate colony counting was 7.48 × 10^9^ CFU.

### 3.4. Immobilization Effect of W33 in Different Carriers

**Figure 4:**
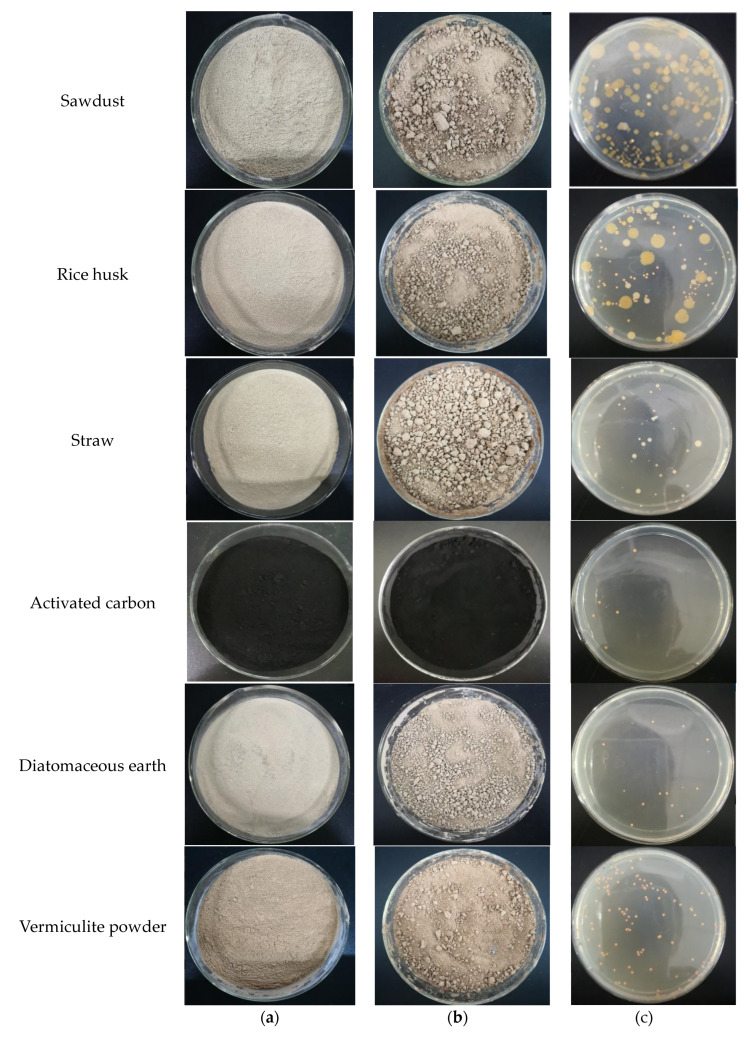
Immobilization of W33 with six carriers. (**a**) Initial carriers; (**b**) immobilized bacterial agents; and (**c**) bacterial agent 10^6^ diluted plates.

In our study, sawdust, rice husk, diatomaceous earth, straw, activated carbon, and vermiculite powder were used as alternative immobilization carriers. Some carriers contain a large amount of biomass, so they are easily infected with other bacteria during the immobilization process. This will result in competition with the immobilized major bacteria, which is not conducive to immobilization. In addition, each bacterium has its own suitable microenvironment, and different carriers have different biological affinities for microorganisms, which will have an important impact on the density of the immobilized bacteria. We investigated the two above characteristics of each carrier and selected the best biological carrier from them. Samples of immobilized bacterial agents prepared from these carrier materials are shown in Figure 4. The trend of infection with other bacteria during immobilization can be seen in Figure 4c.

The W33 immobilization rates of the different carriers are shown in Table 1, which reflects the biological affinity of different carriers for W33. Though rice husk has the highest immobilization rate of W33, it contains a lot of other mixed bacteria, which is not conducive to the growth of W33 (Figure 4c). It can be seen that only vermiculite powder guaranteed the high immobilization rate of W33, and it did not infect other bacteria during immobilization. As an immobilized carrier, vermiculite powder can not only ensure the good purity of major bacteria, but it also has a high biological affinity. Therefore, vermiculite powder was selected as the immobilized carrier for the next study.

### 3.5. Biodegradation Analysis of Petroleum-Contaminated Soil between the Different W33 Bacterial Agents

Standard curve of absorbance–petroleum concentration: the standard curve equation of absorbance at the wavelength of 254 nm (Y) and petroleum concentration (X) is: Y = 75.112 X − 0.0904.

**Figure 5:**
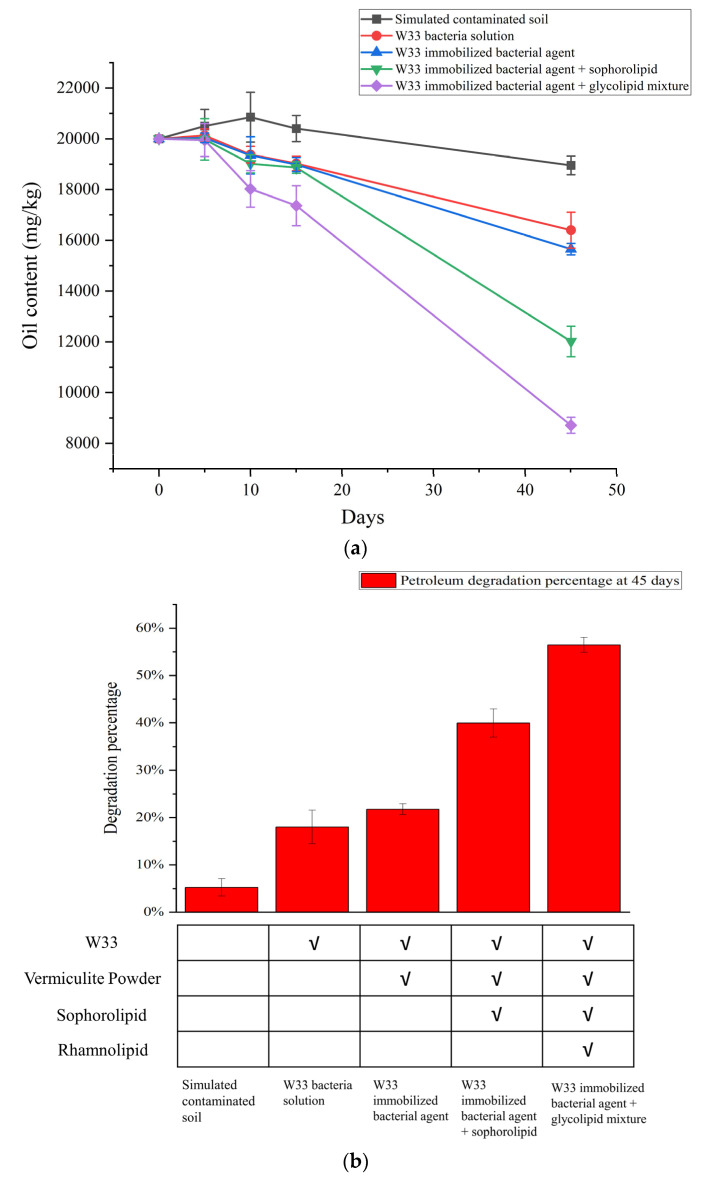
Degradation results of the petroleum-contaminated soil. (**a**) Petroleum degradation curve of each simulated contaminated soil group within 45 days and (**b**) histogram of the degradation rate of each group at 45 days. The experiments were performed in triplicate, and the error bars represent the standard deviations.

The biodegradation abilities of the five biodegradation groups, (1) the simulated contaminated soil with the W33 bacteria solution added, (2) the simulated contaminated soil with the W33-immobilized bacterial agent added, (3) the simulated contaminated soil with the W33-immobilized bacterial agent and sophorolipids added, (4) the simulated contaminated soil with the W33-immobilized bacterial agent and glycolipid mixture added, and (5) the simulated contaminated soil as the control group, were tested. Samples of the contaminated soil were taken on days 5, 10, 15, and 45, and the residual petroleum content was determined. The average of the 3 parallel samples was used to calculate the petroleum degradation rate. The petroleum degradation measurement results were collected and the degradation curves are shown in Figure 5a. After 45 days of biodegradation, compared with 18.1% of the W33 bacteria solution group, 21.6% of the W33-immobilized bacterial agent group, and 40.1% of the W33-immobilized bacterial agent + sophorolipid group, 56.3% of the petroleum was removed from the 20,000 mg/kg simulated contaminated soil in the group with the W33-immobilized bacterial agent + glycolipid mixture. As for the approximately 5% degradation percentage in the simulated contaminated soil in the control group, we speculate that this was due to the loss of volatile substances in the petroleum during weathering and the biodegradation of indigenous microorganisms in the system (Figure 5b). The group with the W33-immobilized bacterial agent + glycolipid mixture reflected a significant advantage, with the average degradation rate reaching 250.2 mg/d. It can be seen that W33 has a good petroleum degradation ability, and the W33-immobilized bacterial agent + glycolipid mixture has absolute advantages compared with the other immobilization methods in this study.

### 3.6. Analysis of the Microscopic Appearance of Each Petroleum Degradation Soil Group under an Electron Microscope

**Figure 6:**
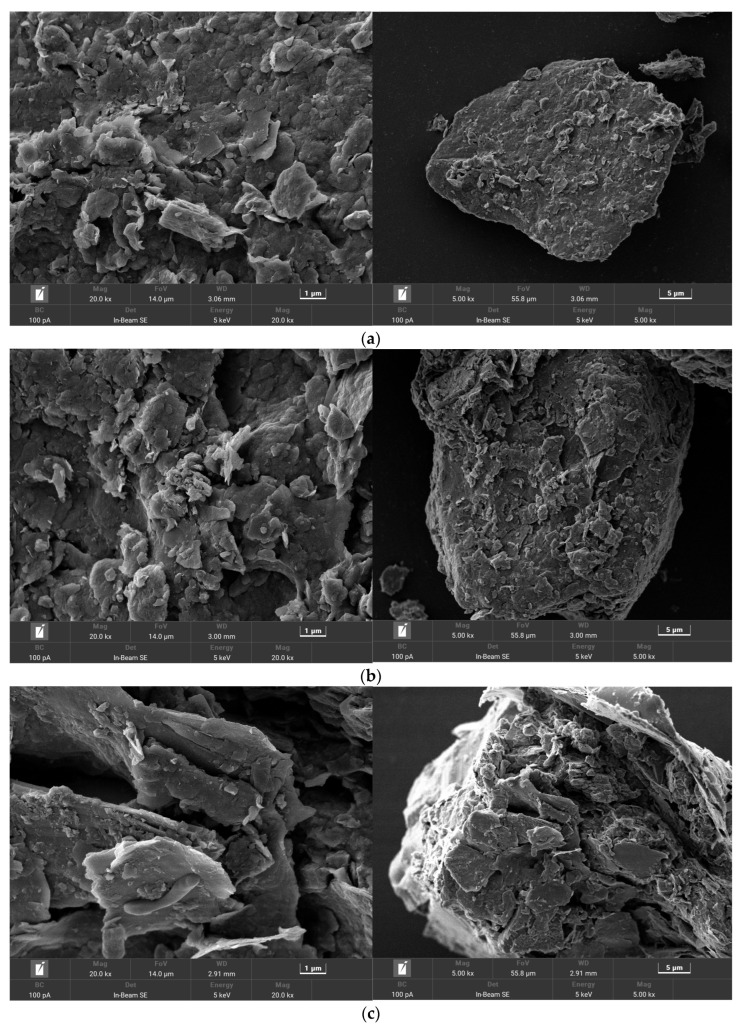

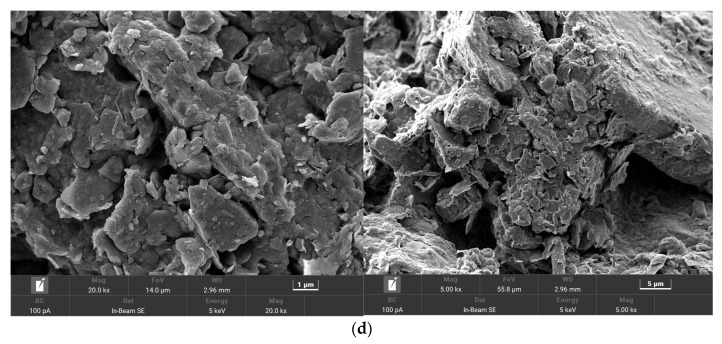
Electron microscope photos of the soil samples of the selected petroleum degradation groups: (**a**) the simulated contaminated soil; (**b**) the simulated contaminated soil with the W33 bacteria solution added; (**c**) the simulated contaminated soil with the W33-immobilized bacterial agent added; and (**d**) the simulated contaminated soil with the W33-immobilized bacterial agent with the sophorolipid and rhamnolipid mixture added.

In order to further explain the improvement in the petroleum degradation performance caused by the W33-immobilized bacterial agent, we selected (1) the simulated contaminated soil group as the control; (2) the W33 bacteria solution group; (3) the W33 vermiculite powder immobilized bacterial agent group; and (4) the W33-immobilized bacterial agent + sophorolipid and rhamnolipid mixture group as the representation. The soil samples of the four representative groups were taken at 45 days and observed microscopically under an electron microscope, and the pictures are shown in Figure 6.

As shown in Figure 6a, no microorganisms were found on the soil surface of the simulated contaminated soil group, and the surface of the soil particles is relatively regular and flat. Figure 6b shows that no spherical or rod-shaped W33 bacterial cells with a size of about 3 μm appear after adding the W33 bacterial solution to the simulated contaminated soil; this is possibly due to the number of surviving W33 cells being too small and the selection of the visual field being limited. In Figure 6c,d, rod-shaped microbial cells can be clearly seen attached to the soil surface, and the surface of the soil sample particles has a loose and porous structure, resulting in an increase in the surface area which allows the same volume of soil to accommodate more microbial cells to survive, which may be the reason why the W33-immobilized inoculum has a higher degradation efficiency than the W33-free bacterial solution. Yet the W33-immobilized bacterial agent with the sophorolipid and rhamnolipid mixture group showed no significant difference with the W33-immobilized bacterial agent group in the electron microscope photos. In addition, after 45 days of degradation, we counted the W33 colonies using the dilution spread plate method to observe the number of W33 surviving cells in each group. The results obtained are shown in Table 2.

## 4. Discussion

With the great development of modern industry, our demand for energy is increasing, which is followed by environmental pollution and destruction. Petroleum will inevitably pollute soil in the process of exploitation, transportation, and utilization. With the development of biotechnology, bioremediation of oil-polluted soil by microorganisms has become a feasible option. In this paper, the study on high-density fermentation and immobilization of *Gordonia alkanivorans* W33 has made a beneficial attempt regarding its bioremediation in oil-contaminated soil.

We screened the key factors of W33 fermentation by conducting a single-factor design experiment. As the concentration of Ca^2+^ added to the medium increased, the biomass increased by 14.66% at 0.06 mM (Figure S1a), which was the maximum increase in biomass. This may be due to the fact that the addition of trace amounts of Ca^2+^ makes the intracellular Ca^2+^ flow better between the calmodulin-like proteins, thereby making calmodulin-like activation or induced biochemical reactivity higher. There was no direct evidence that calmodulin is also present in prokaryotes, but so many studies have indicated that there is a class of proteins in the prokaryote that are similar in terms of their structure and properties to calmodulin in eukaryotes–calmodulin-like proteins [43]. It can not only participate in the activation of phosphodiesterase, NAD kinase, and adenylate cyclize but also induce the proliferation process of some prokaryotic microorganisms and accelerate the rate of their division [47]. The tendency of biomass changes due to the change in Mg^2+^ concentration is consistent with the addition of Ca^2+^ during the culture of W33. At an Mg^2+^ concentration of 0.09 mM, the maximum increase in biomass was 21.66% (Figure S1b). This indicates that Mg^2+^ also plays an important role in W33 cells, except that the enzymes in many metabolic pathways are magnesium-dependent, such as isocitrate lyase; the same is true for many enzymes involved in nuclei acid chemistry [46]. Due to the close combination of Mg^2+^ and water molecules, many enzymes with a cofactor of Mg^2+^ are actually transported to a specific catalytic site after the combination of the water and Mg^2+^; then, the catalytic reaction begins. For example, when many ATP hydrolases with Mg^2+^ as a cofactor catalyze the reaction, water molecules are carried by Mg^2+^ to the central part of the enzymatic reaction; at the same time, the phosphodiester bond is destroyed to complete the hydrolysis of ATP and the transfer of phosphate groups [45].

We predicted the best set of fermentation process parameters by using software and used it in 5 L fermenter fermentation. The results of 5 L fermentation show that the high cell density fermentation technology can significantly increase the fermentation unit in the fermentation broth, and the number of viable cells increased by 175% compared with conventional batch fermentation. The CFU of W33 increases significantly under our fermentation conditions, and the increase in bacterial concentration will also increase the activity of enzymes that degrade petroleum hydrocarbons in the fermentation broth, thereby increasing the degradation rate of solid waste.

In our study of W33 immobilization, only vermiculite powder guarantees a higher concentration of W33 in the immobilized bacterial agent; it is not infected by other bacteria during immobilization. The expanded vermiculite powder has a high specific surface area and porosity and can accommodate more microorganisms during immobilization. At the same time, it can effectively improve the air circulation of the soil after it is added to the soil as a filler. Vermiculite powder also has good adsorption properties. In addition to being used to adsorb heavy metal ions in polluted waters [48], it can also be used as a filler for soil and water moisturization. The above characteristics lead to the fact that vermiculite powder is more suitable for the immobilization of bacteria W33 than other immobilization carriers. As for the other carriers we chose, sawdust, rice husk, diatomaceous earth, straw, and activated carbon, although they have been proven to be effective microbial immobilization carriers in previous studies [31,32,33,34,35], the results achieved in this study were not as good as vermiculite powder. In particular, sawdust and rice husk may introduce additional lignin in bioremediation, causing secondary pollution.

During the bioremediation process, vermiculite powder can have a protective effect on W33 through its pore space, provide protection for soil microorganisms [49], and prevent predation by other organisms in the soil. In addition, it can also reduce the toxicity of the polluted environment to microorganisms, thereby enhancing the degradation activity of microorganisms [12]. These can explain that the petroleum degradation effect of the W33-immobilized bacterial agent group is better than that of the W33-free bacteria solution group. Surfactants can emulsify and disperse petroleum into a degrading environment for utilization by microorganisms. At present, most of the surfactant components in the oil spill dispersants used in the oil pollution treatment process are chemical surfactants, e.g., model oil dispersant Corexit EC9500A, Slickgone LTSW, Ardrox 6120, and Finasol OSR52, which can easily cause secondary pollution [50,51,52,53]. The biosurfactants used in this study are sophorolipids and rhamnolipids, which are a class of lipid substances produced by microorganisms such as bacteria and yeast and are similar in structure to chemical surfactants, containing both hydrophilic and hydrophobic groups in their molecular structure. Compared with traditional chemical surfactants, biosurfactants not only have the properties of surfactants, but also have the advantages of low toxicity, good stability, and easy biodegradation. It has been shown that the use of biosurfactants to prepare oil spill dispersants not only has good petroleum dispersing effects but also promotes the growth of petroleum-hydrocarbon-degrading bacteria, which greatly promotes the degradation of crude oil [54]. Most of the existing related reports are added to the formula in the form of a mixture of various surfactants [55,56]. Lu et al. found that gradually increasing the proportion of rhamnolipids in the mixed system of sophorolipids and rhamnolipids would reduce the minimum surface tension in the oil spill system, and they had a lower critical micelle concentration (CMC) than a single glycolipid. In addition, when the mass ratio of rhamnolipids and sophorolipids was 0.9, the binary mixed system had the lowest CMC, and it was determined that the emulsification rate reached the highest when the mass ratio of application agent to oil was 0.25:1 [36]. The best results in each experimental group in this study used a mass ratio of rhamnolipids to sophorolipids of 0.9 and a mass ratio of agent oil of 0.25:1, and the degradation efficiency was significantly higher than that of the W33-free bacterial solution group, the W33-immobilized bacterial agent group, and the W33-immobilized bacterial agent with the single sophorolipid group.

In general, a key factor involved in the lack of biodegradation success has been the rapid decline in the size of populations of active cells to levels ineffective to achieve the objective, following the introduction of microorganisms into the soil [49]. As Table 2 shows, after 45 days of degradation, the simulated contaminated soil with the W33-immobilized bacterial agent with the mixed glycolipids added has the highest quantity of W33 surviving cells, and the simulated contaminated soil with the W33-immobilized bacterial agent added was second. This suggested that in addition to the immobilized carrier of vermiculite powder providing a better living environment for the W33 bacteria, the addition of the biosurfactant sophorolipids and rhamnolipids further improved the survival rate of the W33 bacteria during the biodegradation process. The possible reason is that the mixture of sophorolipids and rhamnolipids reduces the surface tension and viscosity of the petroleum, enhances the fluidity of the petroleum, promotes mass transfer between the petroleum and microorganisms, and is convenient for being contacted by W33 cells and used as a carbon source.

In summary, in this work, we successfully increased the cell concentration of *Gordonia alkanivorans* W33 by using our high-density fermentation method and demonstrated that the W33-immobilized bacterial agent + glycolipid mixture has a good petroleum degradation ability. However, further larger scale in situ repair experiments are needed to prove the effectiveness of this method.

## 5. Conclusions

The study shows that increasing bacteria concentrations through high-density fermentation and immobilizing bacteria with vermiculite powder, combined with the addition of a sophorolipid and rhamnolipid mixture, can effectively repair oil-contaminated soil. Compared with other carriers, vermiculite powder shows better adsorption performance and a lower rate of infection with other mixed bacteria during immobilization. On this basis, the addition of the biosurfactant sophorolipid and rhamnolipid mixed system further greatly improved the degradation efficiency of the W33-immobilized bacterial agent on petroleum in simulated contaminated soil. The degradation rate of the W33-immobilized bacterial agent + glycolipid mixture reached 56.3% in the soil with 20,000 mg/kg petroleum content in only 45 days, and the average degradation rate reached 250.2 mg/kg/d. The high petroleum degradation efficiency of the W33-immobilized bacterial agent with the mixture of sophorolipids and rhamnolipids shows its broad promotion and application prospects.

## Figures and Tables

**Table 1:** Effects of the immobilization carriers on the immobilization rate of W33.

Type of Carrier	Immobilization Rate of W33 (%)
Rice husk	152
Vermiculite powder	82.6
Sawdust	56.2
Diatomaceous earth	20.6
Activated carbon	7.25
Straw	0.873

Immobilization rate = total number of immobilized bacteria/total number of bacteria added. The data were the average of three parallel experiments.

**Table 2:** W33 colony counting results of the soil samples diluted and spread on culture plates. The data are the average of three parallel experiments.

Simulated Contaminated Soil	Simulated Contaminated Soil with the W33 Bacteria Solution added	Simulated Contaminated Soil with theW33-Immobilized Bacterial Agent added	Simulated Contaminated Soil with the W33-Immobilized Bacterial Agent with the Mixed Glycolipids added
0	3.0 × 10^8^	5.8 × 10^9^	2.6 × 10^10^

The data are the average of three parallel experiments.

## Data Availability

Not applicable.

## Supplementary Materials

The following supporting information can be downloaded at: https://www.mdpi.com/article/10.3390/bioengineering10050561/s1, Figure S1: The effect of adding inorganic salts on the biomass of W33.; Figure S2: Fitness of predictive value and experimental value of final biomass; Table S1: Response surface test factor level table; Table S2: Response surface test plan design and results; Table S3: Results of variance analysis.
