# Supplementary material for: Efficient Bioremediation of Petroleum-Contaminated Soil by Immobilized Bacterial Agent of Gordonia alkanivorans W33
Source: Bioengineering (Basel). 2023 May 8;10(5):561. doi: 10.3390/bioengineering10050561 (PMC10215891; doi:10.3390/bioengineering10050561)
Supplement: Supplementary file 1 [file bioengineering-10-00561-s001.zip › bioengineering-2322018-supplementary.pdf]

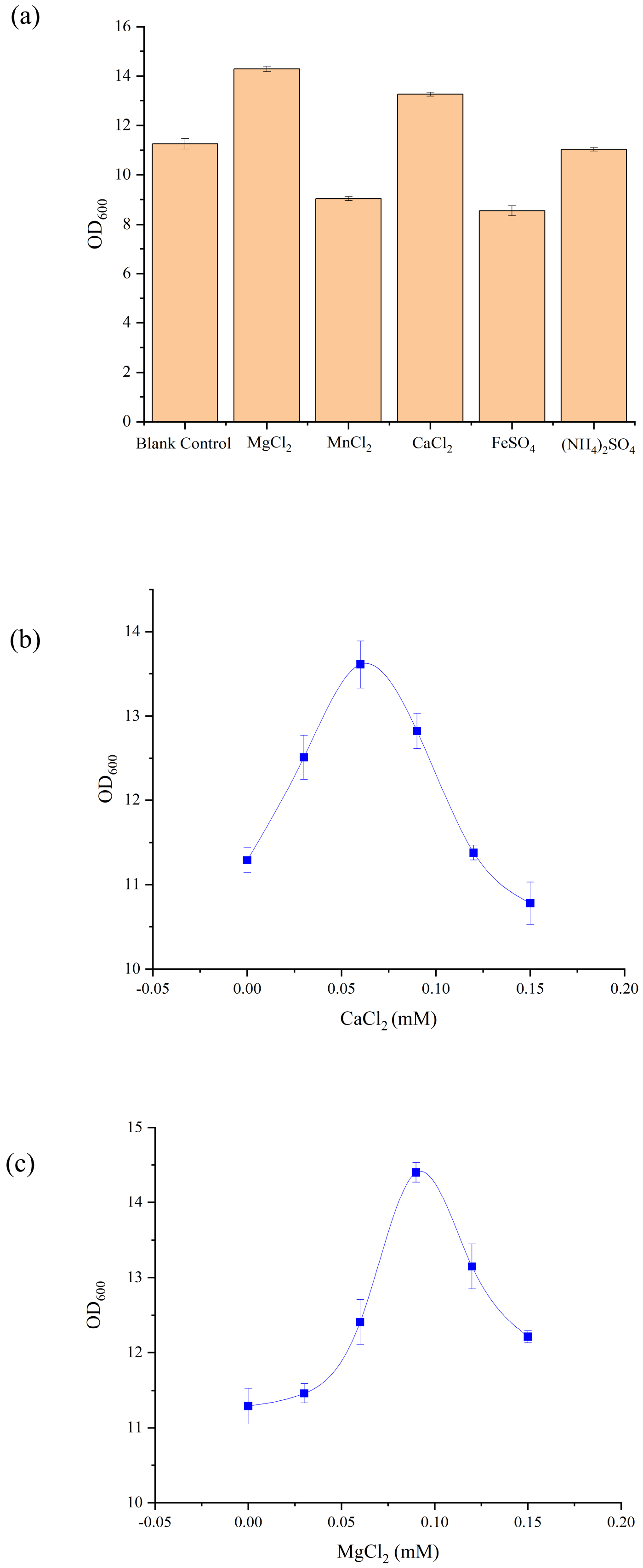
(a)
OD_{600}
16
14
12
10
8
6
4
2
0
Blank Control
$MgCl_2$
$MnCl_2$
$CaCl_2$
$FeSO_4$
$(NH_4)_2SO_4$
(b)
OD_{600}
14
13
12
11
10
-0.05
0.00
0.05
0.10
0.15
0.20
$CaCl_2$ (mM)
(c)
OD_{600}
15
14
13
12
11
10
-0.05
0.00
0.05
0.10
0.15
0.20
$MgCl_2$ (mM)

(d)

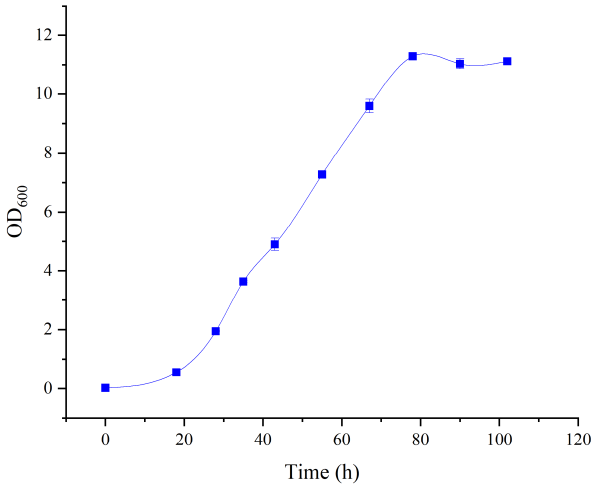

Figure S1. The effect of adding inorganic salts on the biomass of W33. (a) Effect of adding 0.06mM $MgCl_2$, $MnCl_2$, $CaCl_2$, $FeSO_4$ and $(NH_4)_2SO_4$ alone on W33 biomass; (b) Effect of $CaCl_2$ concentration on W33 biomass; (c) Effect of $MgCl_2$ concentration on W33 biomass; (d) W33 growth curve without adding inorganic salts.

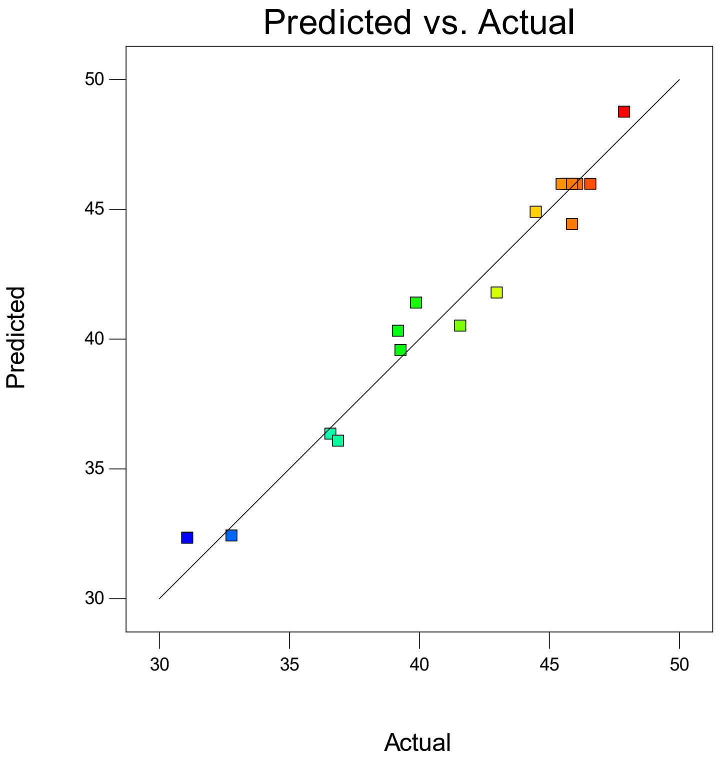

Figure S2. Fitness of predictive value and experimental value of final biomass

Table S1. Response surface test factor level table

Level	Factor		
	Time（A）/h	$MgCl_2$（B）/mM	$CaCl_2$（C）/mM
-1	72	0.06	0.03
0	80	0.09	0.06
1	88	0.12	0.09

Table S2. Response surface test plan design and results

Run	A	B	C	Y
1	1	1	0	44.5
2	1	0	1	43.0
3	1	0	-1	39.2
4	0	1	-1	45.9
5	0	0	0	46.6
6	1	-1	0	36.6
7	-1	-1	0	32.8
8	-1	0	1	41.6
9	0	0	0	46.1
10	0	1	1	47.9
11	0	-1	-1	36.9
12	-1	1	0	39.3
13	-1	0	-1	31.1
14	0	0	0	45.7
15	0	0	0	45.5
16	0	-1	1	39.9
17	0	0	0	45.9

Table S3. Results of variance analysis

Source of variance	Sum of square	Degree of freedom	Mean square	F-Value	p-Value	Significant
Model	400.53	9	44.5	25.12	0.0002	**
A-Time	42.78	1	42.78	24.15	0.0017	**
B-concentration of $MgCl_2$	123.24	1	123.24	69.58	< 0.0001	**
C concentration of $CaCl_2$	46.56	1	46.56	26.29	0.0014	**
AB	0.49	1	0.49	0.28	0.6152	
AC	11.22	1	11.22	6.34	0.04	*
BC	.25	1	0.25	0.14	0.7183	
A^2	141.28	1	141.28	79.76	< 0.0001	**
B^2	14.68	1	14.68	8.29	0.0237	*
C^2	8.76	1	8.76	4.95	0.0615	
Residual	12.4	7	1.77			
Missing item	11.69	3	3.9	21.89	0.0061	**
Pure error	0.71	4	0.18			
Total deviation	412.93	16				

Note: “**” means very significant (p<0.01), “*” means significant (0.01<p<0.05)
